# Heterogeneity in Immune Cell Content in Malignant Pleural Mesothelioma

**DOI:** 10.3390/ijms19041041

**Published:** 2018-03-30

**Authors:** Jorien Minnema-Luiting, Heleen Vroman, Joachim Aerts, Robin Cornelissen

**Affiliations:** Erasmus MC Cancer Institute, Department of Pulmonary Medicine, ’s-Gravendijkwal 230, 3015 CE Rotterdam, The Netherlands; j.minnema-luiting@erasmusmc.nl (J.M.-L.); h.vroman@erasmusmc.nl (H.V.); j.aerts@erasmusmc.nl (J.A.)

**Keywords:** malignant pleural mesothelioma (MPM), tumor microenvironment (TME), heterogeneity, immunotherapy, myeloid-derived suppressor cells (MDSCs), tumor-associated macrophages (TAMs), tumor-infiltrating lymphocytes (TIL), regulatory T cells (Tregs)

## Abstract

Malignant pleural mesothelioma (MPM) is a highly aggressive cancer with limited therapy options and dismal prognosis. In recent years, the role of immune cells within the tumor microenvironment (TME) has become a major area of interest. In this review, we discuss the current knowledge of heterogeneity in immune cell content and checkpoint expression in MPM in relation to prognosis and prediction of treatment efficacy. Generally, immune-suppressive cells such as M2 macrophages, myeloid-derived suppressor cells and regulatory T cells are present within the TME, with extensive heterogeneity in cell numbers. Infiltration of effector cells such as cytotoxic T cells, natural killer cells and T helper cells is commonly found, also with substantial patient to patient heterogeneity. PD-L1 expression also varied greatly (16–65%). The infiltration of immune cells in tumor and associated stroma holds key prognostic and predictive implications. As such, there is a strong rationale for thoroughly mapping the TME to better target therapy in mesothelioma. Researchers should be aware of the extensive possibilities that exist for a tumor to evade the cytotoxic killing from the immune system. Therefore, no “one size fits all” treatment is likely to be found and focus should lie on the heterogeneity of the tumors and TME.

## 1. Introduction

Malignant pleural mesothelioma (MPM) is a rare and highly aggressive cancer arising from the mesothelial cells of the pleura with a median survival of 9 months. More than 70 percent of MPM results from exposure of asbestos [1]. The only licensed treatment is palliative antifolate and platinum combination chemotherapy which results in a moderate overall survival benefit of about three months [2]. MPM consists of three histological variants: (1) epithelioid (~60% of mesotheliomas); (2) sarcomatoid, characterized by spindle cell morphology (~20% of mesotheliomas); (3) biphasic, a mixture of epithelioid and sarcomatoid characteristics (~20% of mesotheliomas) [3,4]. Currently, accepted prognosticators include stage and histology of which sarcomatoid subtype results in the lowest survival rates [5]. It has been demonstrated that protumor and antitumor immune responses within the tumor and associated stroma also correlate with the clinical outcome of MPM [6,7]. This review discusses current knowledge of heterogeneity in immune cell content in MPM in relation to prognosis and prediction of treatment efficacy.

## 2. Tumor Microenvironment (TME) in Mesothelioma

The mesothelioma tumor microenvironment (TME) is a complex and heterogeneous mixture of stromal, endothelial and immune cells. This composition differs between individuals and histologic types, and can change upon administered anti-tumor therapies [8]. The role of immune cells within the TME has become a major area of interest, as these immune cells are capable of influencing tumor growth. In general, immune infiltration in tumors include natural killer (NK) cells, B and T lymphocytes, mast cells, neutrophils, myeloid-derived suppressor cells (MDSCs), macrophages and dendritic cells (DCs). NK cells, cytotoxic T cells, mature DCs and T helper cells are known to be anti-tumorgenic, while others, like regulatory T cells (Tregs), type 2 macrophages, and MDSCs suppress the immune response and therefore favor tumor growth and dissemination [9]. The TME in mesothelioma is unique as it arises from exposure of mesothelial cells to asbestos fibers [6,8]. It is known to be highly immunosuppressive, with higher numbers of immunosuppressive cells such as type 2 tumor associated macrophages and Tregs [10,11,12,13].

## 3. Macrophages

Macrophages are specialized phagocytic cells which play a dual role in cancer depending on their differentiation. Schematically, tumor-associated macrophages (TAMs) can be divided into classically activated (M1) macrophages and alternatively activated (M2) macrophages. M1 macrophages have pro-inflammatory, tissue destructive and anti-tumor activity. Whereas M2 macrophages can be seen as pro-tumorgenic by promoting the metastatic capacity of a tumor due to production of multiple cytokines (e.g., IL-1, IL-6, IL-10, VEGF and TGF-β). TAMs derive from circulating monocytic precursors. In tumors, chemokines play an important role in recruitment of monocytes. Once recruited, interleukins such as IL-4, IL-13 and IL-10 produced by tumor infiltrating lymphocytes (TILs) promote differentiation of macrophages towards an M2 phenotype [14,15]. Certain drugs can skew M2 macrophages into a more M1 phenotype [16,17]. Table 1 describes the antibodies and their associated immune cells. Burt et al. performed a CD68 staining on tissue microarray of 52 MPM patients. Macrophages were abundantly present in both epithelial (*n* = 34) and non-epithelial (*n* = 18) mesothelioma (tumor infiltrating macrophages in percentage of tumor area (%) 25.2 ± 9.3 and 29.7 ± 10.2, *p* = 0.11). The relatively high standard deviation indicates large heterogeneity in macrophage infiltration in MPM. In seven patients, three with epithelial and four with non-epithelial MPM, flow cytometry was performed displaying high levels of CD163 and CD206, characterizing them as M2 macrophages. The absolute number of macrophages was associated with worse prognosis in non-epithelioid mesothelioma after surgery, but not in epithelioid mesotheliomas [18]. Cornelissen et al. described expression of CD68 and CD163 in tumor specimens of sixteen patients with epithelial MPM, eight of them receiving induction chemotherapy and surgery and eight patients receiving chemotherapy only. In both groups macrophages were abundantly present, whereby a large spreading in actual number of macrophages was seen (surgery vs. non-surgery 211.3/0.025 cm^2^ ± 80.2 and 213.9/0.025 cm^2^ *±* 100.4 *p* = 1.0). Most of these macrophages showed a M2 phenotype. A higher percentage of M2 macrophages was significantly negative correlated with overall survival [19]. In lung cancer, Cornelissen et al. described ten MPM patients with local tumor outgrowth after surgery and their matched controls without local tumor outgrowth. Two biphasic and eighteen epithelial MPM patients were included. Macrophage infiltration was characterized by large heterogeneity with a mean macrophage count of 202/0.025 cm^2^, ranging from 45 to 408/0.025 cm^2^. These macrophages show a M2 phenotype with a mean count of 153/0.025 cm^2^ and a range of 42 to 422/0.025 cm^2^ [20]. Marcq et al. found macrophages in stroma of all 54 MPM specimens, with a majority of samples having less than 50% CD68+ cells. Numbers of stromal macrophages were positively correlated to the number of stromal Tregs (*R* = 0.41, *p* = 0.002), suggesting that macrophages stimulate and recruit CD4+ cells by affecting the adaptive immune response [15,21]. Schürch et al. found heavy infiltration of M2 macrophages in all 40 MPM analyzed [22]. Table 2 summarizes the extent of macrophage infiltration found in these studies.

## 4. Myeloid-Derived Suppressor Cells

Myeloid cells are abundantly present in stroma of MPM [8]. MDSCs are immature myeloid cells with immune suppressive capacities. MDSCs are generally characterized by being positive for CD33 and CD11b and low or negative for HLA-DR. They induce Tregs and produce nitric oxide and arginase, leading to loss of function of CD4+ and CD8+ T cells. These strongly immunosuppressive characteristics promote immune escape, tumor growth, invasion and angiogenesis [8,18]. Immune suppression by MDSCs was found to be one of the main factors for immunotherapy insufficiency [10]. MDSCs are induced by several tumor-derived factors, e.g., prostaglandins. Celecoxib reduces prostaglandin levels. Veltman et al. found celecoxib to improve dendritic cell-based immunotherapy by reducing numbers of MDSCs and suppressing function [10]. In mice, MDSCs are defined by IL-4Rα expression [23]. Burt et al. found IL-4Rα to be highly expressed on tumor cells of 52 MPM specimens, with presence of IL-4Rα in 97% of epithelial and 95% of non-epithelial tumors. Only a scattered and small fraction of stromal cells stained positive for IL-4Rα, conversely macrophages were predominantly found in stroma [24]. In another study of Burt et al., flow cytometry was performed on mononuclear cell suspensions from seven MPM patients; these macrophages displayed high levels of IL-4Rα [18]. Awad et al. found myeloid cells (CD33) to represent approximately 42% of CD45+ immune cells (range 5.7–86.1%); 0.6–31% of these myeloid cells were typed as MDSCs [25].

## 5. T Cells and Natural Killer Cells in Mesothelioma

TILs play an important role in the immune defense in cancer. They recognize tumor-specific antigens presented on HLA-1, to then kill the tumor cells via production of perforins and granzymes. In many cancers, T cell infiltration is associated with a good prognosis [26,27,28]. T helper CD4+ cells play an important role in the generation of a T cell-mediated antitumor response, via stimulation of CD8+ TILs and NK cells and via activation of antigen-presenting cells (APCs) [29,30,31]. NK cells are lymphoid cells of the innate immune system with strong immunostimulatory effector functions and efficient cytotoxic capacity [32]. In 1982, Leigh et al. were the first to describe a relation between presence of significant lymphoid infiltration and prolonged survival in 58 mesothelioma patients. Tumors were found without, with insignificant and with significant lymphocyte inflammation. Due to absence of modern immunohistochemical agents, no lymphocyte subsets could be identified [33]. Mudhar et al. performed immunohistochemical staining on fifteen cases of epithelioid MPM, scoring CD45, CD3, CD20 and CD56 with 0 (no significant infiltrate), 1 (non-brisk) or 2 (brisk infiltrate). In one patient, none of these immune cells were present. Specimens demonstrated some heterogeneity in numbers of T lymphocyte and NK cells. With brisk infiltration of T-lymphocytes and NK cells in one case, non-brisk infiltration in eleven and ten cases, respectively. The other three and four specimens showed absence of T lymphocytes and NK cells, respectively. No B cells were present in any specimens. No relation was found between the infiltration of immune cells and survival [34]. A comprehensive analysis by Hegmans et al. demonstrated leukocyte infiltration in all four MPM patients. Most inflammatory cells were identified as macrophages and NK cells (CD16). Some heterogeneity was noted. Eosinophils, mast cells, B cells and neutrophils were rarely detected. DCs were not found in the biopsies [35]. Immunohistochemical analysis of T cells of 32 extrapleural pneumonectomy specimens after induction chemotherapy was performed by Anraku et al. Results are summarized in Table 3. The distribution of T cells varies, with only CD3+ and CD45RO+ TILs showing normal distribution. The coefficient of variation ranges from 0.49 to 0.87, implying substantial heterogeneity. In multivariate data analyses, presence of CD8+ TILs was associated with better prognosis [36].

Yamada et al. [37] analyzed presence of TILs and NK cells in 44 MPM cases, comprised of 26 epithelioid, fourteen biphasic and four sarcomatoid mesotheliomas. Results of T cell subtype counts are presented in Table 4. Again, the heterogeneity is substantial, indicated by wide ranges and CVs ranging from 0.82 to 1.54. Presence of CD4+ and CD8+ T cells was strongly correlated (*R* = 0.74, and *p* = 0.001). In multivariate data analysis high CD8+ TILs and epithelioid histology were independent favorable prognostic factors [37].

Awad et al. [25] performed flow cytometry with various leukocyte markers on 38 malignant mesothelioma, with all histologies. They found considerable variability in immune cell infiltration across tumors. Numbers of CD45+ leukocytes were increased in non-epithelioid mesothelioma compared to epithelioid mesothelioma (median 91.4% vs. 64.1%). Amount of T cells ranged from 5.2% to 81.2% of CD45+ cells, with a higher fraction of T cells in non-epithelioid mesothelioma. There was considerable variability in numbers of leukocytes and in immune cell composition across cases [25]. Marcq et al. [21] found lymphocytic infiltration in all 54 tested mesotheliomas, ranging from 20% to 80% of stromal cells. The fourteen chemotherapy pretreated samples showed higher numbers of lymphocytes. CD8+ TILs were the predominant cell type of the immune infiltrate and were present in all samples. In 70% of the untreated and 57% of the pretreated samples, the majority of the lymphocytes were CD8+ TILs. High expression of CD45RO on stromal lymphocytes was associated with worse response to chemotherapy. T helper cells were found in 85% of untreated and 100% of pretreated samples. T helper cells in lymphoid infiltrates were associated with better survival in multivariate analysis [21]. Suzuki et al. [38] evaluated inflammatory responses in tumor and stroma of 175 chemotherapy naive epithelioid MPM specimens with H&E-stained slides. Acute response was represented by presence of neutrophils, while chronic inflammation was represented by lymphocytes and plasma cells. Acute inflammatory reaction was sparse in tumors and stroma, with high scores (>1% of total area) in 18% of specimens. The chronic reaction was more heterogenic, with high scores (>50% of total area) in 37% of tumors and 34% of stromal tissue. In multivariate analysis, chronic inflammation in stroma was an independent predictor of survival while other inflammatory responses were not significantly correlated with survival [38]. These studies suggest considerable infiltration of TILs in mesothelioma. Higher levels of TILs are associated with better survival in most studies.

## 6. Regulatory T Cells (Tregs)

FOXP3+CD25+CD4+ regulatory T cells maintain self-tolerance and prevent autoimmune disease. They are abundantly present in tumors, where they suppress activation and proliferation of effector T cells. High numbers of Tregs are associated with poor prognosis in many cancers [39]. Hegmans et al. demonstrated that human mesothelioma biopsies harbor significant numbers of Tregs at the rim of the tumor [35]. Marcq et al. [21] found Tregs to be present in 72% of samples, both chemotherapy pretreated and untreated. Lower numbers of Tregs were seen in samples pretreated with cisplatin and pemetrexed [21]. DeLong et al. performed flow cytometry on malignant pleural effusions from seven patients with mesothelioma; 7.8% ± 6.8% of T-lymphocytes were functionally suppressive CD4+CD25+ cells, which might be Tregs. This is a significant lower number of Tregs than seen in malignant effusions secondary to breast cancer or NSCLC. Some heterogeneity was noted, including two patients with <3% CD4+CD25+ T cells and one patient with 21% CD4+CD25+ T cells in pleural effusion. The latter was a sarcomatoid subtype [40].

## 7. B Lymphocytes

B lymphocytes contribute to humoral immunity as they can differentiate into antibody-secreting plasma cells. Also, B cells can stimulate T cells or serve as APCs. In several cancers, including mesothelioma, B lymphocyte infiltration is associated with better patient survival [41]. Two studies found low numbers of B lymphocytes (CD20) in mesothelioma [34,35]. A third study found low B lymphocyte (CD19) infiltration (median 3% of CD45+ cells), although some outliers with B cell infiltration up to 51.8% of CD45+ cells were seen [26]. Patil et al. [42] classified three molecular subgroups based on immune profiles; in one subgroup high numbers of B cells were found [42]. Generally, B cell infiltration in mesothelioma is sparse, although a subgroup with higher numbers of B cells is described. More research is needed for determining the clinical implications.

## 8. Cancer-Associated Fibroblasts (CAFs)

The major component of the TME are cancer-associated fibroblasts, also known as tumor-associated fibroblasts [43]. MPM recruit and activate CAFs by secreting fibroblast growth factor-2 (FGF-2) and platelet-derived growth factor-AA (PDGF-AA) [44]. CAFs can contribute to tumor growth by inhibiting cytotoxic T cell influx and by secreting several growth factors such as hepatocyte growth factor, thereby inducing angiogenesis [44,45]. In 1996 Harvey et al. demonstrated infiltration of CAFs in six of eight MPM samples [46]. Li et al. performed histological analyses on specimens from 51 MPM patients and revealed considerable CAF infiltration [44].

## 9. PD-L1 Expression and Other Immune Checkpoints

Programmed cell death 1 (PD-1) is an immune checkpoint receptor present on activated T cells. PD-1 and its ligands, PD-L1 and PD-L2, which are expressed by tumor cells and/or stromal cells share immunosuppressive capacities [47]. In several tumors, including NSCLC, PD-L1 enrichment is associated with higher response rates to PD-1 and PD-L1-blocking antibodies [47,48,49]. However, responses have also been observed in PD-L1-negative patients [50]. We found eight studies evaluating PD-L1 expression in mesothelioma. A summary of the results is displayed in Table 5. PD-L1 was found to be expressed in 16% to 65% of malignant mesothelioma. PD-L1 expression is higher in non-epithelioid mesothelioma compared to epithelioid mesothelioma (37.5–97.4% vs. 6.7–31%) [21,25,42,50,51,52,53,54]. Several studies found higher PD-L1 expression to be an independent prognostic indicator for worse overall survival in multivariate data analysis [50,52,53,55]. Khanna et al. [54] analyzed PD-L1 expression in peritoneal and pleural fluid of respectively six and three mesothelioma patients. PD-L1 expression was found in all samples, varying from 12% to 83%. Immune cells were evaluated for PD-1 expression in seven samples. PD-1 was expressed in 21.8% of CD4+ cells and 37.5% of CD8+ cells. Together, these data suggest that malignant effusions of mesothelioma patients have high PD-L1 expression on tumor cells as well as PD-L1 and PD-1 on infiltrating immune cells [54]. Staining for other checkpoint inhibitors such as TIM-3 and LAG-3 was performed by Marcq et al. [21] TIM-3 expression was found in 36 of 54 samples (both treated and untreated). LAG-3 expression was absent in all 54 MPM samples, pointing out the possible opportunities of TIM-3 as a promising immunotherapy target in mesothelioma. In multivariate analysis, TIM-3 expression in lymphoid aggregates was a prognosticator for better survival [21].

## 10. Discussion

We performed a comprehensive literature search focusing on the heterogeneity of immune cell infiltration, PD-L1 expression and other immune checkpoints in MPM. The composition of TME holds therapeutic and prognostic implications [6,7]. Stage and histology are currently accepted prognostic indicators [5], but evidence is accumulation that infiltrating immune cells and expression of immune checkpoints are of high prognostic value in MPM [7,50,51,52,53]. Infiltration of M2 macrophages seems to be associated with worse prognoses [18,19,20], as is PD-L1 expression [50,51,52,53]. Infiltration of cytotoxic T cells was associated with better prognosis in MPM in most studies [21,33,36,37,38].

TME composition differs between various histologic subtypes and individuals [25]. Macrophages are found to be abundantly present in all MPM, although the level of infiltration can vary significantly. Macrophages generally show an M2 phenotype [18,19,20,21,22,24]. Stroma of MPM is infiltrated by MDSCs [8,18,25]. Leukocyte infiltration was found in almost all mesothelioma, with higher numbers of leukocytes in non-epithelioid mesothelioma [25]. T cell subsets showed considerable heterogeneity with wide ranges and high coefficients of variation across all studies. Cytotoxic T cells, NK cells and T helper cells were most abundantly present [21,33,34,35,36,37,38]. B cell infiltration is sparse, although a (molecular) subgroup with an increased number B cells is described [25,34,35,42]. Significant numbers of Tregs were found in biopsies and pleural fluid of mesothelioma [21,35,40]. Tumor growth promoting CAFS are found in TME of most MPM [44,46]. PD-L1 expression is commonly found in MPM, with higher expression in non-epithelioid histologic subtypes [21,25,42,50,51,52,53,54].

Altogether, substantial heterogeneity in immune cell content in mesothelioma was found. MPM are highly infiltrated by immune effector cells, but also immune suppressive cells such as Tregs and M2 macrophages and PD-L1 expression are found. Apparently, the tumor finds several ways to bypass the immune system. Thoroughly mapping the composition of the TME is rational in targeting therapy in mesothelioma. For example, tumors with high amounts of T effector cells and Tregs might benefit from a combination of immunotherapy and drugs that control Tregs, to invigorate immunotherapy efficacy. Tumors highly infiltrated by MDSCs might benefit more from (dendritic cell-based) immunotherapy when this is combined with celecoxib, as this reduces the suppressive function and number of MDSCs [10]. In MPM expressing PD-L1 and cytotoxic T cells present in TME, treatment with PD-(L)1 inhibitors is more rational. Other rational treatment options include nintedanib or emactuzumab for the skewing of M2 macrophages to the M1 subtype in TME highly infiltrated with M2 macrophages [16,17], or OX40 for the stimulation of cytotoxic T cells when they are not already present in the TME [56]. Inhibition of the cytokines FGF-2, PDGF-AA, and HGF may be appropriate in MPM infiltrated with CAFs [44]. This opens up a whole new era of personalized immunotherapy in which we are just scratching the surface. Researchers should be aware of the extensive possibilities that exist for a tumor to evade the cytotoxic killing by the immune system. Therefore, no “one size fits all” treatment is likely to be found and focus should lie on the heterogeneity of the tumors and TME.

## Figures and Tables

**Table 1:** Cell surface markers and correlating immune cell type.

Surface Marker	Present on
CD3	T lymphocytes
CD4	T helper cells
CD8	Cytotoxic T cells
CD11b	Monocytes, macrophages, MDSCs, NK cells, eosinophils, neutrophils, basophils, dendritic cells, mast cells, CD8+ T cells, B cells
CD16	Natural killer cells, myeloid cells, monocytes, neutrophils
CD19	B cells
CD20	B cells
CD33	Myeloid cells
CD45	Leucocytes
CD45RO	T effector and memory cells
CD56	Natural killer cells
CD68	Macrophages
CD163	M2 macrophages
CD206	M2 macrophages
Foxp3+CD4+CD25+	Regulatory T cells

**Table 2:** Infiltration of TAMs and M2 macrophages in mesothelioma.

Study	*n*	CD68+	Coefficient of Variation (CV) *	CD163+	Coefficient of Variation (CV) *
[18]	52	25.2 ± 9.3%, (epithelial) 29.7 ± 10.2%, (non-epithelial)	0.37, (epithelial) 0.34, (non-epithelial)	n.a. ***	n.a. ***
[19]	16	211.3 ** ± 80.2, (surgery) 213.9 ** ± 100.4, (non-surgery)	0.37, (surgery) 0.47, (non-surgery)	168.3 ** ± 80.2, (surgery) 164.1 ** ± 82.5, (non-surgery)	0.48, (surgery) 0.50, (non-surgery)
[20]	20	202 **	Range 45–408	153 **	Range 42–422
[21]	54	Present in all specimens	n.a. ***	n.a. ***	n.a. ***
[22]	40	Heavy infiltration	n.a. ***	Heavy infiltration	n.a. ***

* CV is defined as the ratio of the standard deviation to the mean; ** Cell count per field; *** n.a is not applicable.

**Table 3:** Infiltration of T cell subtypes in 32 extrapeural pneumonectomy specimens.

Surface Marker	Mean (Cell Count per Field)	Standard Deviation	Coefficient of Variation (CV) *
CD3+	232.16	114.1	0.49
CD4+	119.9	94.2	0.79
CD8+	73.1	40.2	0.55
CD25+	17.5	12.6	0.72
FOXP3+	21.8	19.0	0.87
CD45RO+	115.7	56.2	0.49

* CV is defined as the ratio of the standard deviation to the mean.

**Table 4:** Infiltration of T cell subtypes in 44 MPM cases [37].

Surface Marker	Mean (Cell Count per Field)	Standard Deviation	Coefficient of Variation (CV) *	Range	Median
CD4+	51.1	41.8	0.82	0.2–159.7	37.3
CD8+	103.3	106.9	1.03	8.8–547.5	64.5
CD56+	5.4	8.3	1.54	0.0–41.8	1.8

* CV is defined as the ratio of the standard deviation to the mean.

**Table 5:** PD-L1 expression in mesothelioma.

Study	PD-L1 Antibody	*n*	Positivity (%)	PD-L1 Positive (*n* (%))	PD-L1 Positive in Epithelioid (*n* (%))	PD-L1 Positive in Non-Epithelioid (*n* (%))	Survival in PD-L1+ (Months)	Survival in PD-L1- (Months)	*p* Value
[53]	5H1-A3	106	≥5	42 (40)	14/68 (21)	37/38 (97)	5	14.5	<0.0001
[51]	E1L3N	77	>1%	16 (21)	7/53 (13)	9/24 (38)	4.8	16.3	0.012
[26]	E1L3N	39	≥1%	18 (46)	8/26 (31)	10/13 (77)	shorter	longer	0.15
[50]	E1L3N	58	≥1%	17 (29)	8/34 (24)	9/24 (38)	n.a.*	n.a.*	n.a.*
[50]	SP142	58	≥1%	10 (17)	4/34 (12)	6/24 (25)	4	13	0.016
[54]	rabbit	65	≥5%	41 (63)	n.a.*	n.a.*	23.0	33.3	0.35
[21]	SP142	54	≥1%	35 (65)	n.a.*	More in sarcomatoid	n.a.*	n.a.*	n.a.*
[52]	E1L3N	175	≥5%	57 (33)	46/148 (31)	11/27 (41)	6	18	<0.01
[42]	SP142	99	>1%	16 (16)	5/75 (6.7)	9/24 (38)	shorter	longer	

* n.a is not applicable.

## References

[1] Carbone M., Ly B.H., Dodson R.F., Pagano I., Morris P.T., Dogan U.A., Gazdar A.F., Pass H.I., Yang H. (2012). Malignant mesothelioma: Facts, myths, and hypotheses. J. Cell. Physiol..

[2] Vogelzang N.J., Rusthoven J.J., Symanowski J., Denham C., Kaukel E., Ruffie P., Gatzemeier U., Boyer M., Emri S., Manegold C. (2003). Phase III study of pemetrexed in combination with cisplatin versus cisplatin alone in patients with malignant pleural mesothelioma. J. Clin. Oncol..

[3] Attanoos R.L., Gibbs A.R. (1997). Pathology of malignant mesothelioma. Histopathology.

[4] Travis W.D., Brambilla E., Burke A.P., Marx A., Nicholson A.G. (2015). Introduction to The 2015 World Health Organization Classification of Tumors of the Lung, Pleura, Thymus, and Heart. J. Thorac. Oncol..

[5] Sugarbaker D.J., Flores R.M., Jaklitsch M.T., Richards W.G., Strauss G.M., Corson J.M., DeCamp M.M., Swanson S.J., Bueno R., Lukanich J.M. (1999). Resection margins, extrapleural nodal status, and cell type determine postoperative long-term survival in trimodality therapy of malignant pleural mesothelioma: Results in 183 patients. J. Thorac. Cardiovasc. Surg..

[6] Mossman B.T., Shukla A., Heintz N.H., Verschraegen C.F., Thomas A., Hassan R. (2013). New insights into understanding the mechanisms, pathogenesis, and management of malignant mesotheliomas. Am. J. Pathol..

[7] Bograd A.J., Suzuki K., Vertes E., Colovos C., Morales E.A., Sadelain M., Adusumilli P.S. (2011). Immune responses and immunotherapeutic interventions in malignant pleural mesothelioma. Cancer Immunol. Immunother..

[8] Yap T.A., Aerts J.G., Popat S., Fennell D.A. (2017). Novel insights into mesothelioma biology and implications for therapy. Nat. Rev. Cancer.

[9] Hegmans J.P., Aerts J.G. (2014). Immunomodulation in cancer. Curr. Opin. Pharmacol..

[10] Veltman J.D., Lambers M.E., van Nimwegen M., Hendriks R.W., Hoogsteden H.C., Aerts J.G., Hegmans J.P. (2010). COX-2 inhibition improves immunotherapy and is associated with decreased numbers of myeloid-derived suppressor cells in mesothelioma. Celecoxib influences MDSC function. BMC Cancer.

[11] Veltman J.D., Lambers M.E.H., van Nimwegen M., Hendriks R.W., Hoogsteden H.C., Hegmans J.P., Aerts J.G. (2010). Zoledronic acid impairs myeloid differentiation to tumour-associated macrophages in mesothelioma. Br. J. Cancer.

[12] Veltman J.D., Lambers M.E.H., van Nimwegen M., de Jong S., Hendriks R.W., Hoogsteden H.C., Aerts J.G., Hegmans J.P. (2010). Low-dose cyclophosphamide synergizes with dendritic cell-based immunotherapy in antitumor activity. J. Biomed. Biotechnol..

[13] Van der Most R.G., Currie A.J., Mahendran S., Prosser A., Darabi A., Robinson B.W.S., Nowak A.K., Lake R.A. (2009). Tumor eradication after cyclophosphamide depends on concurrent depletion of regulatory T cells: A role for cycling TNFR2-expressing effector-suppressor T cells in limiting effective chemotherapy. Cancer Immunol. Immunother..

[14] Mantovani A., Sozzani S., Locati M., Allavena P., Sica A. (2002). Macrophage polarization: Tumor-associated macrophages as a paradigm for polarized M2 mononuclear phagocytes. Trends Immunol..

[15] Solinas G., Germano G., Mantovani A., Allavena P. (2009). Tumor-associated macrophages (TAM) as major players of the cancer-related inflammation. J. Leukoc. Biol..

[16] Pradel L.P., Ooi C.-H., Romagnoli S., Cannarile M.A., Sade H., Rüttinger D., Ries C.H. (2016). Macrophage Susceptibility to Emactuzumab (RG7155) Treatment. Mol. Cancer Ther..

[17] Herrmann F., Ayaub E., Parthasarathy P., Ackermann M., Inman M.D., Kolb M.R.J., Wollin L., Ask K., Tandon K. (2017). Nintedanib attenuates the polarization of profibrotic macrophages through the inhibition of tyrosine phosphorylation on CSF1 receptor. Am. J. Respir. Crit. Care Med..

[18] Burt B.M., Rodig S.J., Tilleman T.R., Elbardissi A.W., Bueno R., Sugarbaker D.J. (2011). Circulating and tumor-infiltrating myeloid cells predict survival in human pleural mesothelioma. Cancer.

[19] Cornelissen R., Lievense L.A., Maat A.P., Hendriks R.W., Hoogsteden H.C., Bogers A.J., Hegmans J.P., Aerts J.G. (2014). Ratio of intratumoral macrophage phenotypes is a prognostic factor in epithelioid malignant pleural mesothelioma. PLoS ONE.

[20] Cornelissen R., Lievense L.A., Robertus J.-L., Hendriks R.W., Hoogsteden H.C., Hegmans J.P., Aerts J.G. (2015). Intratumoral macrophage phenotype and CD8+ T lymphocytes as potential tools to predict local tumor outgrowth at the intervention site in malignant pleural mesothelioma. Lung Cancer.

[21] Marcq E., Siozopoulou V., De Waele J., van Audenaerde J., Zwaenepoel K., Santermans E., Hens N., Pauwels P., van Meerbeeck J.P., Smits E.L.J. (2017). Prognostic and predictive aspects of the tumor immune microenvironment and immune checkpoints in malignant pleural mesothelioma. Oncoimmunology.

[22] Schürch C.M., Forster S., Brühl F., Yang S.H., Felley-Bosco E., Hewer E. (2018). The “don’t eat me” signal CD47 is a novel diagnostic biomarker and potential therapeutic target for diffuse malignant mesothelioma. Oncoimmunology.

[23] Mandruzzato S., Solito S., Falisi E., Francescato S., Chiarion-Sileni V., Mocellin S., Zanon A., Rossi C.R., Nitti D., Bronte V. (2009). IL4Rα+ Myeloid-Derived Suppressor Cell Expansion in Cancer Patients. J. Immunol..

[24] Burt B.M., Bader A., Winter D., Rodig S.J., Bueno R., Sugarbaker D.J. (2012). Expression of Interleukin-4 Receptor Alpha in Human Pleural Mesothelioma Is Associated with Poor Survival and Promotion of Tumor Inflammation. Clin. Cancer Res..

[25] Naito Y., Saito K., Shiiba K., Ohuchi A., Saigenji K., Nagura H., Ohtani H. (1998). CD8+ T cells infiltrated within cancer cell nests as a prognostic factor in human colorectal cancer. Cancer Res..

[26] Zhang L., Conejo-Garcia J.R., Katsaros D., Gimotty P.A., Massobrio M., Regnani G., Makrigiannakis A., Gray H., Schlienger K., Liebman M.N. (2003). Intratumoral T cells, recurrence, and survival in epithelial ovarian cancer. N. Engl. J. Med..

[27] Schumacher K., Haensch W., Röefzaad C., Schlag P.M. (2001). Prognostic significance of activated CD8(+) T cell infiltrations within esophageal carcinomas. Cancer Res..

[28] Zhu J., Paul W.E. (2008). CD4 T cells: Fates, functions, and faults. Blood.

[29] Friedman K.M., Prieto P.A., Devillier L.E., Gross C.A., Yang J.C., Wunderlich J.R., Rosenberg S.A., Dudley M.E. (2012). Tumor-specific CD4+ melanoma tumor-infiltrating lymphocytes. J. Immunother..

[30] Neurath M.F., Finotto S. (2012). The emerging role of T cell cytokines in non-small cell lung cancer. Cytokine Growth Factor Rev..

[31] Van Acker H.H., Capsomidis A., Smits E.L., Van Tendeloo V.F. (2017). CD56 in the Immune System: More Than a Marker for Cytotoxicity?. Front. Immunol..

[32] Leigh R.A., Webster I. (1982). Lymphocytic infiltration of pleural mesothelioma and its significance for survival. S. Afr. Med. J..

[33] Mudhar H.S., Wallace W.A.H. (2002). No relationship between tumour infiltrating lymphocytes and overall survival is seen in malignant mesothelioma of the pleura. Eur. J. Surg. Oncol..

[34] Hegmans J.P.J.J. (2006). Mesothelioma environment comprises cytokines and T-regulatory cells that suppress immune responses. Eur. Respir. J..

[35] Anraku M., Cunningham K.S., Yun Z., Tsao M.-S., Zhang L., Keshavjee S., Johnston M.R., de Perrot M. (2008). Impact of tumor-infiltrating T cells on survival in patients with malignant pleural mesothelioma. J. Thorac. Cardiovasc. Surg..

[36] Yamada N., Oizumi S., Kikuchi E., Shinagawa N., Konishi-Sakakibara J., Ishimine A., Aoe K., Gemba K., Kishimoto T., Torigoe T. (2010). CD8+ tumor-infiltrating lymphocytes predict favorable prognosis in malignant pleural mesothelioma after resection. Cancer Immunol. Immunother..

[37] Awad M.M., Jones R.E., Liu H., Lizotte P.H., Ivanova E.V., Kulkarni M., Herter-Sprie G.S., Liao X., Santos A.A., Bittinger M.A. (2016). Cytotoxic T Cells in PD-L1-Positive Malignant Pleural Mesotheliomas Are Counterbalanced by Distinct Immunosuppressive Factors. Cancer Immunol. Res..

[38] Suzuki K., Kadota K., Sima C.S., Sadelain M., Rusch V.W., Travis W.D., Adusumilli P.S. (2011). Chronic inflammation in tumor stroma is an independent predictor of prolonged survival in epithelioid malignant pleural mesothelioma patients. Cancer Immunol. Immunother..

[39] Nishikawa H., Sakaguchi S. (2014). Regulatory T cells in cancer immunotherapy. Curr. Opin. Immunol..

[40] DeLong P., Carroll R.G., Henry A.C., Tanaka T., Ahmad S., Leibowitz M.S., Sterman D.H., June C.H., Albelda S.M., Vonderheide R.H. (2005). Regulatory T cells and cytokines in malignant pleural effusions secondary to mesothelioma and carcinoma. Cancer Biol. Ther..

[41] Ujiie H., Kadota K., Nitadori J., Aerts J.G., Woo K.M., Sima C.S., Travis W.D., Jones D.R., Krug L.M., Adusumilli P.S. (2015). The tumoral and stromal immune microenvironment in malignant pleural mesothelioma: A comprehensive analysis reveals prognostic immune markers. Oncoimmunology.

[42] Patil N.S., Righi L., Koeppen H., Zou W., Izzo S., Grosso F., Libener R., Loiacono M., Monica V., Buttigliero C. (2018). Molecular and Histopathological Characterization of the Tumor Immune Microenvironment in Advanced Stage of Malignant Pleural Mesothelioma. J. Thorac. Oncol..

[43] Baglole C.J., Ray D.M., Bernstein S.H., Feldon S.E., Smith T.J., Sime P.J., Phipps R.P. (2006). More than structural cells, fibroblasts create and orchestrate the tumor microenvironment. Immunol. Investig..

[44] Li Q., Wang W., Yamada T., Matsumoto K., Sakai K., Bando Y., Uehara H., Nishioka Y., Sone S., Iwakiri S. (2011). Pleural Mesothelioma Instigates Tumor-Associated Fibroblasts To Promote Progression via a Malignant Cytokine Network. Am. J. Pathol..

[45] Lo A., Wang L.-C., Scholler J., Monslow J., Avery D., Newick K., O’Brien S., Evans R.A., Bajor D.J., Clendenin C. (2015). Tumor-Promoting Desmoplasia Is Disrupted by Depleting FAP-Expressing Stromal Cells. Cancer Res..

[46] Harvey P., Warn A., Newman P., Perry L.J., Ball R.Y., Warn R.M. (1996). Immunoreactivity for hepatocyte growth factor/scatter factor and its receptor, met, in human lung carcinomas and malignant mesotheliomas. J. Pathol..

[47] Gandini S., Massi D., Mandalà M. (2016). PD-L1 expression in cancer patients receiving anti PD-1/PD-L1 antibodies: A systematic review and meta-analysis. Crit. Rev. Oncol. Hematol..

[48] Fehrenbacher L., Spira A., Ballinger M., Kowanetz M., Vansteenkiste J., Mazieres J., Park K., Smith D., Artal-Cortes A., Lewanski C. (2016). Atezolizumab versus docetaxel for patients with previously treated non-small-cell lung cancer (POPLAR): A multicentre, open-label, phase 2 randomised controlled trial. Lancet.

[49] Rittmeyer A., Barlesi F., Waterkamp D., Park K., Ciardiello F., von Pawel J., Gadgeel S.M., Hida T., Kowalski D.M., Dols M.C. (2017). Atezolizumab versus docetaxel in patients with previously treated non-small-cell lung cancer (OAK): A phase 3, open-label, multicentre randomised controlled trial. Lancet.

[50] Combaz-Lair C., Galateau-Sallé F., McLeer-Florin A., Le Stang N., David-Boudet L., Duruisseaux M., Ferretti G.R., Brambilla E., Lebecque S., Lantuejoul S. (2016). Immune biomarkers PD-1/PD-L1 and TLR3 in malignant pleural mesotheliomas. Hum. Pathol..

[51] Cedrés S., Ponce-Aix S., Zugazagoitia J., Sansano I., Enguita A., Navarro-Mendivil A., Martinez-Marti A., Martinez P., Felip E. (2015). Analysis of Expression of Programmed Cell Death 1 Ligand 1 (PD-L1) in Malignant Pleural Mesothelioma (MPM). PLoS ONE.

[52] Inaguma S., Lasota J., Wang Z., Czapiewski P., Langfort R., Rys J., Szpor J., Waloszczyk P., Okoń K., Biernat W. (2018). Expression of ALCAM (CD166) and PD-L1 (CD274) independently predicts shorter survival in malignant pleural mesothelioma. Hum. Pathol..

[53] Mansfield A.S., Roden A.C., Peikert T., Sheinin Y.M., Harrington S.M., Krco C.J., Dong H., Kwon E.D. (2014). B7-H1 expression in malignant pleural mesothelioma is associated with sarcomatoid histology and poor prognosis. J. Thorac. Oncol..

[54] Khanna S., Thomas A., Abate-Daga D., Zhang J., Morrow B., Steinberg S.M., Orlandi A., Ferroni P., Schlom J., Guadagni F. (2016). Malignant Mesothelioma Effusions Are Infiltrated by CD3+ T Cells Highly Expressing PD-L1 and the PD-L1+ Tumor Cells within These Effusions Are Susceptible to ADCC by the Anti-PD-L1 Antibody Avelumab. J. Thorac. Oncol..

[55] Cedrés S., Ponce-Aix S., Pardo-Aranda N., Navarro-Mendivil A., Martinez-Marti A., Zugazagoitia J., Sansano I., Montoro M.A., Enguita A., Felip E. (2016). Analysis of expression of PTEN/PI3K pathway and programmed cell death ligand 1 (PD-L1) in malignant pleural mesothelioma (MPM). Lung Cancer.

[56] Curti B.D., Kovacsovics-Bankowski M., Morris N., Walker E., Chisholm L., Floyd K., Walker J., Gonzalez I., Meeuwsen T., Fox B.A. (2013). OX40 is a potent immune-stimulating target in late-stage cancer patients. Cancer Res..

